# Egg Quality Assurance Programs and Egg-associated *Salmonella* Enteritidis Infections, United States

**DOI:** 10.3201/eid1010.040189

**Published:** 2004-10

**Authors:** Gerald A. Mumma, Patricia M. Griffin, Martin I. Meltzer, Chris R. Braden, Robert V. Tauxe

**Affiliations:** * Centers for Disease Control and Prevention, Atlanta, Georgia, USA

**Keywords:** Salmonella Enteritidis (S. Enteritidis), Egg Quality Assurance Program (EQAP), state-sponsored EQAP, industry-sponsored EQAP, change-point procedure, research

## Abstract

A *Salmonella enterica* serovar Enteritidis epidemic in the United States began in 1978, spread to much of the country in the following decade, and began declining in 1996. We examined correlations between annual changes in *S*. Enteritidis incidence in humans and introductions of egg quality assurance programs (EQAPs) in some states to reduce *S*. Enteritidis contamination of eggs. Before EQAPs, 62% of the changes in *S*. Enteritidis incidence were higher than the baseline for each state. After EQAPs, 73%–84% of the changes were below the baseline. Regression analysis showed that a 1% increase in the number of eggs produced under an EQAP was associated with a 0.14% decrease in *S*. Enteritidis incidence (p < 0.05). These data indicate that EQAPs probably played a major role in reducing *S*. Enteritidis illness in these states.

## Abstract 2

A 1% increase in eggs produced under egg quality assurance programs was associated with a 0.14% decrease in *Salmonella* Enteritidis incidence.

An epidemic of infections caused by *Salmonella enterica* serovar Enteritidis in the United States began in New England in 1978 and spread to much of the rest of the country in the next decade. Though the spread has declined in all regions since 1996 (Figure 1), the number and incidence of *S*. Enteritidis infections have not shown substantial decline since 1999 (*1*). Since grade A shell eggs have been implicated as a major source of *S*. Enteritidis infections in humans in the United States (*2*), interventions have been introduced to reduce *S*. Enteritidis infection in poultry and eggs and the resulting illness in humans (*3**–**10*). These interventions include State Egg Quality Assurance Programs (EQAPs), which are voluntary programs that are based on Hazard Analysis Critical Control Point (HACCP) principles and designed around production, management, and monitoring practices to mitigate risk for *S*. Enteritidis contamination of eggs (*3**,**11**,**12*). Motivations for egg producers to adopt an EQAP may include scientific, public health, public relations, or marketing reasons (*13*). Initially, producers enrolled voluntarily into state- or industry-sponsored EQAPs. However, in some states, commercial egg producers are required to participate in EQAPs because egg processors, food commodity brokers, insurance companies, and integrated commercial companies are increasingly demanding producer participation in EQAPs as a condition of egg sales (*12*).

**Figure 1:**
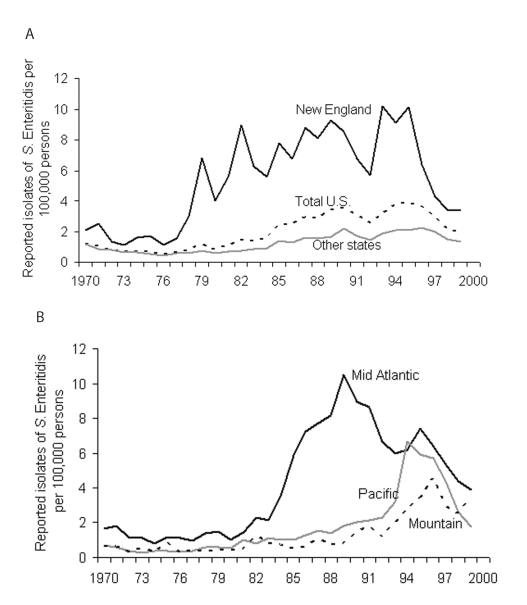
Reported isolates per 100,000 persons of *Salmonella enterica* serovar Enteritidis by region, United States, 1970–1999. A) New England: Connecticut, Maine, New Hampshire, Rhode Island, Vermont. B) Mid-Atlantic: New Jersey, New York, Pennsylvania. Pacific: Alaska, California, Hawaii, Oregon, Washington. Mountain: Arizona, Colorado, Montana, Nevada, New Mexico, Utah. Other states: Alabama, Arkansas, Delaware, Florida, Georgia, Illinois, Indiana, Iowa, Kansas, Kentucky, Louisiana, Maryland, Michigan, Minnesota, Mississippi, Missouri, Nebraska, North Carolina, North Dakota, Ohio, Oklahoma, South Carolina, South Dakota, Tennessee, Texas, Virginia, Washington DC, West Virginia, Wisconsin. Source: Centers for Disease Control and Prevention, National Salmonella Surveillance System (*1*).

Research to date has focused on verifying the role and effectiveness of EQAPs in mitigating *S*. Enteritidis in layer flocks and eggs (*4**,**5**,**13*). Effectiveness might be indicated by reductions of *S*. Enteritidis prevalence in layer flocks (*11**,**14**,**15*), farm environments (*11**,**16*), and eggs produced by infected flocks (*5*) after introducing EQAPs. Reported reductions in *S*. Enteritidis rates in markets with EQAPs have been used to explain the effectiveness of EQAPS in reducing *S*. Enteritidis illness in humans (*2**,**3**,**11**,**16**,**17*). Some evidence shows that interventions that reduce the storage time of shell eggs, internal or ambient temperature, or prevalence of *S*. Enteritidis–positive flocks or that increase diversion of eggs from *S*. Enteritidis-positive flocks to pasteurization, may help reduce the incidence of *S*. Enteritidis in humans (*18*). However, little is known about the contribution of each intervention to the overall reduction in the number of *S*. Enteritidis cases. The present study analyzes flock-based EQAPs to assess their actual contribution to the reduction of *S*. Enteritidis incidence in humans.

## Methods

Baseline incidence was defined as *S*. Enteritidis incidence in the year in which an EQAP was adopted in a state or group of states affected by the *S*. Enteritidis epidemic. We calculated *S*. Enteritidis incidence for a state or group of states as the number of reported human *S*. Enteritidis isolates in a year divided by that state's or group of states' population for that year expressed per 100,000 persons. We defined a state affected by the *S*. Enteritidis epidemic as one for which the *S*. Enteritidis incidence was >1/100,000 in any year between 1980 and 1999.

States that adopted EQAPs were grouped into state- and industry-sponsored EQAPs. We grouped EQAPs into state-sponsored and industry-sponsored types on the basis of whether the state government was actively involved in third-party monitoring, supervision, provision of technical advice, and procedure of handling houses that are found to be *S*. Enteritidis positive. In this study, state-sponsored EQAPs were defined as having active state Department of Agriculture and Department of Health involvement in providing technical advice, supervising and monitoring the programs, requiring third-party auditing, testing eggs for contamination with *S*. Enteritidis if houses were positive, and diverting eggs found to be contaminated with *S*. Enteritidis to pasteurization and hard cooking. Industry-sponsored EQAPs were defined as lacking state government involvement, recommending but not requiring third-party audits of the program, and recommending immediate extra cleaning of *S*. Enteritidis–contaminated houses upon depopulation of the houses (*19*).

We calculated the percentage change in annual *S*. Enteritidis incidence relative to the baseline (hereafter referred to as the change in *S*. Enteritidis incidence):



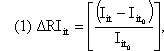



where, I stands for SE incidence; ΔRI for change in SE incidence; i = 1, 2,..., N for state; t = -T,...,-1, 0, 1,..., T for time, t_0_ = year of EQAPs introduction

We then divided the change in *S*. Enteritidis incidence for a given year by the number of years before or after the intervention to get the annualized percentage change in *S*. Enteritidis incidence (hereafter referred to as the annualized change in *S*. Enteritidis incidence). We used two methods to examine changes in *S*. Enteritidis incidence: a simple change-point procedure and regression analysis.

## Change-point Analysis Framework

**Figure 2:**
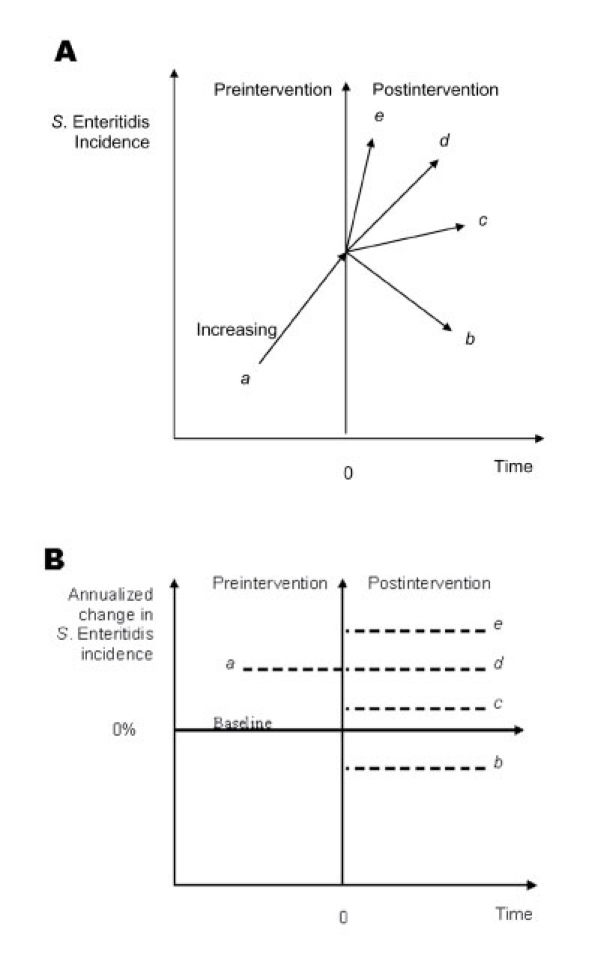
A) Framework to compare *Salmonella enterica* serovar Enteritidis incidence for a given year to the baseline incidence for evidence of intervention effectiveness. Each arrow shows the change of *S*. Enteritidis incidence for a given year, relative to baseline incidence. The letter *a* shows increasing *S*. Enteritidis incidence relative to the baseline incidence, *b* shows a reduction in *S*. Enteritidis incidence relative to the baseline incidence, *c* shows a smaller reduction, *d* shows no change, and *e* shows an increase. B) Framework to compare annualized changes in *S*. Enteritidis incidence for a given year to a baseline incidence for evidence of effectiveness of an intervention. Each dot represents an annualized change in *S*. Enteritidis incidence relative to the baseline change in *S*. Enteritidis incidence, which is 0%. The letter *a* shows preintervention annualized changes in *S*. Enteritidis incidence that are above the baseline, indicating annual increase in incidence; *b* shows postintervention annualized changes in *S*. Enteritidis incidence that are below the baseline; and preintervention rates indicating annual decrease in incidence; *c* are above baseline but below preintervention rates; *d* are above baseline but equal to preintervention rates; and *e* are above preintervention and baseline rates.

We constructed a graph with a horizontal axis representing time in years and a vertical axis representing *S*. Enteritidis incidence (Figure 2A). If an intervention is effective, *S*. Enteritidis incidence should decrease after the baseline year (line b, Figure 2A). If the *S*. Enteritidis incidence had been increasing before the intervention, a smaller increase in incidence after the baseline would also show evidence of effectiveness (line c, Figure 2A). If the *S*. Enteritidis incidence had been decreasing before the intervention, we would expect a faster decrease in incidence after the baseline. Similarly, the lack of change in *S*. Enteritidis incidence observed before, during, and after the intervention would be evidence of lack of effect (line d, Figure 2A), and an increase in *S*. Enteritidis incidence (line e, Figure 2A) would be evidence that the intervention was associated with an acceleration of the epidemic.

The effectiveness of an intervention can further be assessed by using the annualized change in *S*. Enteritidis incidence (Figure 2B). To show evidence of an intervention's effectiveness, we would expect the annualized change in *S*. Enteritidis incidence after intervention to be below the baseline rate (dotted line b, Figure 2B) or the preintervention rate (dotted line b compared to a, Figure 2B). A more modest effect after intervention that shows the epidemic continuing to grow at a diminished annualized rate would find the change in incidence above baseline but below the preintervention level (dotted line c compared to a, Figure 2B). An ineffective intervention would result in an annualized change in *S*. Enteritidis incidence after intervention that is equal to the rate before intervention (dotted line d compared to a, Figure 2B). An annualized change in *S*. Enteritidis incidence above the preintervention rate (line e compared to a, Figure 2B) would be evidence that the intervention was associated with an acceleration of the epidemic. If changes in *S*. Enteritidis incidence continued at about the same rate or were sustained for a number of years, the annualized change in *S*. Enteritidis incidence would, in time, trend toward the baseline. We calculated Yates corrected chi-square values to verify whether changes in observations of annualized *S*. Enteritidis incidence were statistically significant.

We examined observations of pre-EQAP annualized rates of change in *S*. Enteritidis incidence for a period of up to 5 years and for a variable period of up to 8 years of post-EQAP observations. The time periods selected were considered to be long enough to include any relevant lag effects and short enough to exclude confounding influence of other interventions, such as those that require refrigerating eggs. To analyze the timing of the decrease in *S*. Enteritidis incidence, we grouped states on the basis of duration of postintervention follow-up and then type of EQAP and compared the annualized changes in *S*. Enteritidis incidence for each of the 5 years before adopting EQAPs and up to 5 years after adopting EQAPs. For example, states that adopted EQAPs in 1996 had 3 years of common experience with the intervention from 1996 to 1999 and formed a group based on this common length of time. The annualized changes in *S*. Enteritidis provided a measure for comparing incidence of *S*. Enteritidis before, during, and after EQAP adoption.

## Regression Model

We also examined the percentage change in *S*. Enteritidis incidence by using a pooled regression model. The pooling method can be used to combine cross-section and time-series data. This technique allows for the error terms to have equal variance on the chosen values of the explanatory variables within a state, but unequal variance between states (*20**–**25*), which results in efficient and unbiased parameter estimates. We estimated a pooled regression equation for five cross-sectional states (Connecticut, Louisiana, Indiana, Pennsylvania, and California) for 5 years post-EQAP by using SHAZAM (*20*). The pooled regression equation was:



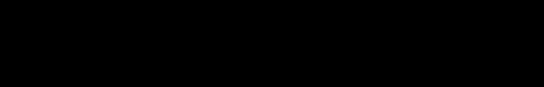



We included Louisiana in the regression model, although it was unaffected by the epidemic, to improve the degrees of freedom for the model. Only four of the states that were affected by the *S*. Enteritidis epidemic had 5 years of post-EQAP experience. Also, we included a dummy variable in the model to control for states that were not affected by the *S*. Enteritidis epidemic.

Independent variables (and types) were percentage of eggs produced in participating farms (continuous), type of EQAP (binary: state- or industry-sponsored: yes/no), number of United States Department of Agriculture (USDA) *S*. Enteritidis outbreak traceback investigations (continuous), proportion of population at high risk for *S*. Enteritidis (children <5 years and seniors >65 years) (continuous), and whether the state was in the northeast geographic region (Connecticut, Pennsylvania, New York) (binary: yes/no). The proportion of eggs produced under EQAPs by state and year was the index to measure participation in EQAPs.

## Data Collection

We sent a detailed questionnaire to state veterinarians and public health officials in all states that were involved with *S*. Enteritidis control and prevention efforts. We also asked state officials to share the questionnaire widely with stakeholders (e.g., state departments of agriculture, laboratory workers, and egg industry officials) in state *S*. Enteritidis mitigation efforts.

The questionnaire collected data on whether egg producers in the state had adopted an EQAP, and if so, the type of EQAP to which most producers in the state adhered (e.g., industry-or state-sponsored), year of EQAP initiation, estimated proportion of total commercial layer-flock participation in the EQAP by year, and elements of the EQAP to which participants were required to adhere. The annual number of *S*. Enteritidis cases was obtained from reports by state and local health departments to the National *Salmonella* Surveillance System (*1*). Estimates of the annual population data for states were obtained from the Bureau of the Census of the U.S. Department of Commerce (*26*).

The annual numbers of eggs produced by state from 1972 to 1999 were obtained from USDA's National Agricultural Statistics Service (NASS) (*27*). To calculate the proportion of eggs produced under an EQAP for each state, we assumed no difference in egg production per layer between layers raised under an EQAP and those raised under no EQAP. We then calculated the proportion of eggs produced under each EQAP as a product of the proportion of total layer flocks that participated in the EQAP and the annual total number of eggs produced by each state. This calculation may overestimate the annual total number of eggs produced for human consumption. The category "table eggs" would provide a closer estimate of eggs produced for human consumption. However, due to confidentiality concerns, NASS does not publish complete information on table egg production.

To estimate a proxy for the proportion of the state's population at high risk for *S*. Enteritidis, we used the resident population <5 years of age and >65 years of age and total resident population. Data for estimates of the resident population by age and state for 1989–1999 were obtained from the U.S. Census Bureau (*26*). We obtained the number of successful *S*. Enteritidis outbreak traceback investigations (investigation to establish origin of *S*. Enteritidis–contaminated eggs) by state from USDA's *S*. Enteritidis Task Force Status Reports for 1990 to 1993 (*8*). Similar information was not available for tracebacks from 1996 to 1999, when the Food and Drug Administration was responsible for tracebacks. The typical procedure when a traceback leads to farms is for the regulatory body to take environmental samples of manure areas, egg belts and escalators, fans, and feed. If the environment tests positive for *S*. Enteritidis, the farmer can either divert the eggs to pasteurization or hard cooking for the lifetime of the flock, divert the eggs until they test negative for *S*. Enteritidis, or depopulate the flock.

## Results

### EQAP

#### Egg Production Under EQAP

We received analyzable results from 41 states. No response was received from Idaho, Maine, Mississippi, New Jersey, New Mexico, Virginia, Washington, Wisconsin, or West Virginia. These states accounted for ≈9% of U.S. shell egg production from 1989 to 1999. State officials in 15 of the 41 states reported that egg producers in their respective states had adopted one of two kinds of EQAPs from 1989 to 1999. Ten (Connecticut, Pennsylvania, California, South Carolina, Maryland, Ohio, Michigan, Utah, New York, Alabama) adopted state-sponsored EQAPs, and 5 (Louisiana, Indiana, Oregon, Florida, Georgia) adopted industry-sponsored EQAPs. Eleven of the 41 responding states were affected by the *S*. Enteritidis epidemic, of which 9 had state-sponsored programs and 2 had industry-sponsored programs. The proportion of eggs produced under EQAPs among the 41 responding states increased from 1% in 1989 to 46% in 1999, and eggs produced under EQAPs among the 11 states that had EQAPs and were affected by the *S*. Enteritidis epidemic increased from 3% in 1989 to 79% in 1999 (Table 1).

**Table 1:** Eggs produced under EQAPs as a percentage of total egg production, 1989–1999^a^

Year	% of eggs produced under EQAPs	
	United States (N = 41)^b^	States with EQAPs affected by *S*. Enteritidis epidemic (N = 11)
1989	1.2	2.5
1990	1.7	2.8
1991	3.6	7.0
1992	5.2	10.5
1993	5.5	10.9
1994	8.6	18.0
1995	10.5	22.2
1996	17.6	38.3
1997	27.0	59.1
1998	34.7	68.8
1999	46.1	78.6

^a^*Salmonella enterica* serovar Enteritidis egg quality assurance programs. ^b^Forty-one states responded to the survey. A state affected by the *S*. Enteritidis epidemic had an *S*. Enteritidis isolation rate >1/100,000 population in any year from 1980 to 1999. States with EQAPs affected by the *S*. Enteritidis epidemic were California, Connecticut, Indiana, Maryland, Michigan, New York, Ohio, Oregon, Pennsylvania, South Carolina, and Utah.

#### Change-point Analysis

**Figure 3:**
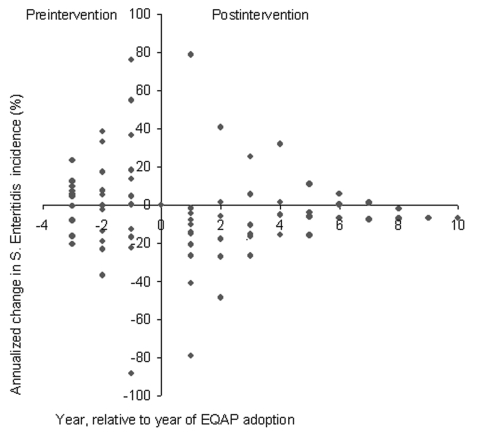
Observed annualized changes in *Salmonella enterica* serovar Enteritidis incidence for 11 states that were affected by the *S*. Enteritidis epidemic and adopted state- or industry-sponsored EQAPS. The 11 states were California, Connecticut, Indiana, Maryland, Michigan, New York, Ohio, Oregon, Pennsylvania, South Carolina, and Utah.

We calculated 55 preintervention and 40 postintervention annualized changes in *S*. Enteritidis incidence for 11 states that were affected by the *S*. Enteritidis epidemic and adopted EQAPs (Table 2). Before adopting any EQAP (state- or industry-sponsored), *S*. Enteritidis incidence relative to the baseline was higher in 62% of the observations and lower in 38% of the observations (Figure 3). After EQAPs were introduced, *S*. Enteritidis incidence increased relative to the baseline in 28% of the post-EQAP observations and decreased in 73% of the observations, which indicates a significant reduction (Yates-corrected chi-square = 9.61, p = 0.0019).

**Table 2:** Annualized rates of change of *Salmonella enterica* serovar Enteritidis incidence before and after adoption of EQAPs in states affected by the epidemic^a,b^

*S*. Enteritidis incidence	With state- or industry- sponsored EQAPs (%)	With state-sponsored EQAPs (%)
Increasing before introduction of EQAPs	34 (62)	28 (62)
Decreasing before introduction of EQAPs	21 (38)	17 (38)
Increasing after introduction of EQAPs	11 (28)	5 (16)
Decreasing after introduction of EQAPs	29 (73)	26 (84)

^a^EQAP, egg quality assurance program. This analysis included 11 states that had state- or industry-sponsored EQAPs and 9 states with state-sponsored EQAPs. Departments of agriculture and health provided technical advice, supervision, and monitoring to state-sponsored EQAPs. State-sponsored required auditing by third parties, tested eggs for contamination with *S*. Enteritidis if layer houses were found to be positive, and diverted eggs found to be contaminated with *S*. Enteritidis to pasteurization and hard cooking. ^b^Percentages may not equal 100% because of rounding.

**Figure 4:**
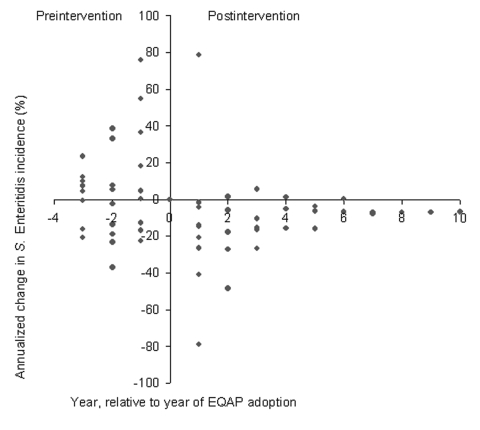
Observed annualized rates of change in *Salmonella enterica* serovar Enteritidis incidence for nine states that were affected by the *S*. Enteritidis epidemic and adopted state-sponsored EQAPS. The nine states were California, Connecticut, Maryland, Michigan, New York, Ohio, Pennsylvania, South Carolina, and Utah.

In the analysis restricted to the nine affected states that adopted state-sponsored EQAPs, we calculated 45 preintervention and 31 postintervention annualized changes (Table 2). *S*. Enteritidis incidence was higher than the baseline in 62% of the pre-EQAP observations and lower in 38% of the observations (Figure 4). After the state-sponsored EQAPs were introduced, *S*. Enteritidis incidence increased relative to the baseline in 16% of the observations and decreased in 84%, which indicates a significant reduction (Yates-corrected chi-square = 14.05, p = 0.00018).

**Figure 5:**
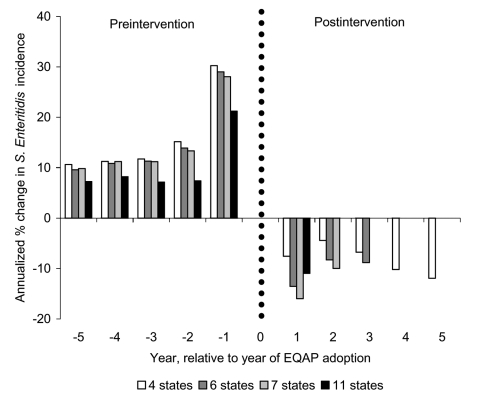
Annualized change in *Salmonella enterica* serovar Enteritidis incidence for groups of states that had egg quality assurance programs (EQAPs) for the same period within group and different periods among groups. The groups of states were 11 states with >1 year of post-EQAP follow-up (Connecticut, Indiana, Pennsylvania, California, South Carolina, Maryland, Ohio, Michigan, Utah, New York, Oregon), 7 states with >2 years of post-EQAP follow-up (Connecticut, Indiana, Pennsylvania, California, South Carolina, Maryland, Ohio), 6 states with >3 years of post-EQAP follow-up (Connecticut, Indiana, Pennsylvania, California, South Carolina, Maryland), and 4 states with >5 years of post-EQAP follow-up (Connecticut, Indiana, Pennsylvania, California).

To analyze the timing of reductions in *S*. Enteritidis incidence, we defined groups of 11, 7, 6, and 4 states with at least 1 year, 2 years, 3 years, and 5 years of post-EQAP follow-up, respectively. n each group, *S*. Enteritidis incidence was increasing before adoption of EQAPs and decreased afterwards. The effect of the intervention was apparent in the first year and was sustained (Figure 5).

#### Results of the Regression Model for States that Adopted EQAPs

Descriptive statistics for variables used in the regression model are presented in Table 3. A 1% increase in the quantity of eggs produced under an EQAP (state- or industry-sponsored) was associated with a 0.14% (p < 0.05) reduction in the change in *S*. Enteritidis incidence (Table 4). A state-sponsored EQAP was associated with a decrease of 72.25% (p < 0.1) in the change in *S*. Enteritidis incidence. A 1% increase in the population at high risk for *S*. Enteritidis was associated with an 8.15% (p < 0.05) increase of the change in *S*. Enteritidis incidence. An increase of 1 in the number of successful USDA *S*. Enteritidis outbreak traceback investigations was associated with an increase of 2.82% (p < 0.001) in the change in *S*. Enteritidis incidence. No significant associations were found for changes in *S*. Enteritidis incidence and states affected by the *S*. Enteritidis epidemic or located in the Northeast region.

**Table 3:** Descriptive statistics for variables in the regression model^a^

Variable	Average	SD
Rate of change in *Salmonella enterica* serovar Enteritidis incidence (%) after EQAP adoption	3.2	44.9
% eggs produced under EQAP by states	63.6	29.5
% a state's population at high risk for *S*. Enteritidis^b^	20.6	0.5
States with state-sponsored EQAP (%)^c^	60	50
States affected by the *S*. Enteritidis epidemic (%)	20	40
Successful *S*. Enteritidis outbreak traceback investigations by state per year^d^	0.3	1.1
States in the northeast region of United States (D)^e^	40	50

^a^EQAP, egg quality assurance program; D, dummy variable. ^b^Children <5 and seniors >65 as a proxy for population at high risk for *S*. Enteritidis. ^c^ Not industry-sponsored EQAP. ^d^Number of *S*. Enteritidis outbreak investigations from United States Department of Agriculture *S*. Enteritidis Task Force Status Reports for 1990 to 1993. ^e^Northeast region includes Connecticut, Pennsylvania, and New York.

**Table 4:** Regression model estimated rates of change in *Salmonella enterica* serovar Enteriditis incidence associated with unit changes in related variables^a^

Explanatory variable	Unit of change	Change in *S*. Enteritidis rate (%)	p value
Intercept^b^		–120.65	< 0.01
Eggs produced under EQAP (%)	1	–0.14	< 0.05
State population at high risk for *S*. Enteritidis ^(%)c^	1	–8.15	< 0.01
State had a state-sponsored EQAP^d^	Yes	–72.25	< 0.1
State was affected by *S*. Enteritidis epidemic^e^	Yes	–3.60	
Successful *S*. Enteritidis outbreak traceback investigations by state per year^f^	Numeral	2.82	< 0.01
State was in the northeast region of the United States^g^	Yes	12.36	

^a^EQAP, egg quality assurance program. ^b^Intercept term is the baseline case, which represents no eggs produced under EQAPs, zero percent of the population at high risk for *S*. Enteritidis, no states affected by *S*. Enteritidis epidemic, no successful outbreak investigations, and all states outside of the northeast region. ^c^Children <5 and seniors >65 years. ^d^Not industry-sponsored EQAP. ^e^A state affected by *S*. Enteritidis epidemic had an isolation rate >1/100,000 persons from 1980 to 1999. ^f^Number of *S*. Enteritidis outbreak investigations from USDA *S*. Enteritidis Task Force status reports for 1990 to 1993. ^g^Northeast region includes Connecticut, Pennsylvania, and New York.

## Discussion and Conclusions

Although a decline in prevalence of *S*. Enteritidis in layer-flock eggs might indicate effectiveness of EQAPs in mitigating *S*. Enteritidis (*4**,**6**,**10**,**12**,**16**,**28*), a connection with reductions of *S*. Enteritidis infections in humans is necessary to indicate effectiveness of the programs in mitigating human illness. Our simple change-point procedure showed a connection between the introduction of EQAPs at the state level and significant reductions in *S*. Enteritidis incidence in humans. The regression analysis found that increasing the quantity of eggs produced under EQAPs was associated with reducing *S*. Enteritidis incidence.

Several factors limited this study. Whether an EQAP was introduced at the beginning of the year or at the end of the year might make a difference, and defining a baseline year might introduce error in the analysis. However, data about the month in which EQAPs were introduced were lacking for most states that adopted these programs. Although some EQAPs are similar in that they were designed through close collaboration among states, they vary in practice and motivation, which limits generalizations about all EQAPs, whether state or industry sponsored. We found verifying the exact practices of each EQAP to be difficult because EQAPs range from self-certification programs, like the 5-Star United Egg Producers program (*19*) practiced in Indiana and Oregon that does not require microbiologic testing for chicks, pullets, layers, and eggs, to the more structured, regulated, rigorous, and costly Pennsylvania Egg Quality Assurance Program (*14*). Eleven of 15 states with EQAPs reported that they required periodic sampling and testing of layer environments, layers, and eggs for *S*. Enteritidis, but 4 (Oregon, Louisiana, Indiana, Georgia) did not. All states that required microbiologic testing, except for Florida, involved their state governments in setting up and monitoring their EQAP programs.

We did not study interactions in the regression model because of few data points and degrees of freedom, which limited the robustness of its results. We were more interested in the direction (positive or negative) of the estimate of the percentage of eggs produced under EQAPs than the magnitude. Also, our results were based on unverified respondent estimates of the proportion of eggs produced under EQAPs, information about the type of EQAP, and when the EQAP was instituted. Further, because accurately estimating prevalence of diabetes, cancer, HIV/AIDS, and pregnancy at the state level was difficult, we used the population of children <5 years of age and seniors >65 years of age for each state to represent the population at high risk for *S*. Enteritidis.

We assumed that eggs produced in a state are applied to meet the consumption needs of that state, and changes in *S*. Enteritidis incidence within the state would reflect the effect of the state's EQAP, but this assumption may not be accurate. Eggs in the United States are distributed widely across the nation through a dynamic system that makes it difficult to track the source and destination of eggs by state. Although data about the source and destination of eggs and egg products are desirable, they are not currently available (*29*).

Not all egg producers immediately join EQAPs, and the percentage of eggs produced in a state under an EQAP varies as producers adopt or leave EQAPs. The simple change-point analysis did not account for these variations and assumed that EQAPs were homogeneous within and among states. The regression model allowed EQAPs to be homogeneous within states and heterogeneous among states.

Our model could not estimate unreported cases in a meaningful way, although these cases constitute most cases of salmonellosis (*14*). The larger proportion of *S*. Enteritidis cases goes unreported (*30*). Other factors may have affected *S*. Enteritidis incidence in humans that we did not account for in this model because of lack of specific data, such as improvements in egg refrigeration during distribution and handling, traceback investigations from 1996 to 1999, and use of pasteurized eggs. However, these measures were not implemented in tandem with the EQAPs within or among states. Therefore, the close temporal association between implementing EQAPs and decreasing rates of *S*. Enteritidis infection indicate the importance of EQAPs as a control strategy.

The results of our study indicate that flock-based interventions have had a positive effect on health by reducing *S*. Enteritidis incidence in humans. These data further indicate that EQAPs probably played a major role in reducing *S*. Enteritidis illness in the United States. Considering that as of 1999, less than half of shell eggs in the United States were produced under EQAPs (Table 1), and that the number of cases and relative rate of *S*. Enteritidis have not shown significant decline since 1999, adopting EQAPS by producers and states would likely improve the public's health and prevent reemergence of egg-based *Salmonella*.
